# Early life microbial exposure and fractional exhaled nitric oxide in school-age children: a prospective birth cohort study

**DOI:** 10.1186/1476-069X-12-103

**Published:** 2013-12-02

**Authors:** Lidia Casas, Christina Tischer, Inge M Wouters, Maties Torrent, Ulrike Gehring, Raquel Garcia-Esteban, Elisabeth Thiering, Dirkje S Postma, Johan de Jongste, Henriëtte A Smit, Alícia Borràs-Santos, Jan-Paul Zock, Anne Hyvärinen, Joachim Heinrich, Jordi Sunyer

**Affiliations:** 1 Centre for Research in Environmental Epidemiology (CREAL), Barcelona, Spain; 2 Hospital del Mar Medical Research Institute (IMIM), Barcelona, Spain; 3 CIBER Epidemiología y Salud Pública (CIBERESP), Barcelona, Spain; 4 Helmholtz Zentrum München, German Research Centre for Environmental Health, Institute of Epidemiology I, Neuherberg, Germany; 5 Institute for Risk Assessment Sciences, Division of Environmental Epidemiology, Utrecht University, Utrecht, The Netherlands; 6 Area de Salud de Menorca, IB-SALUT, Menorca, Spain; 7 Department of Pulmonology, GRIAC research institute, University of Groningen, University Medical Center Groningen, Groningen, The Netherlands; 8 Department of Pediatrics, Division of Respiratory Medicine, Erasmus University Medical Center/Sophia Children’s Hospital, Rotterdam, The Netherlands; 9 Julius Center for Health Sciences and Primary Care, University Medical Center, Utrecht, The Netherlands; 10 Department Environmental Health, National Institute for Health and Welfare, Kuopio, Finland; 11 University Pompeu Fabra, Barcelona, Spain

**Keywords:** Fractional exhaled nitric oxide, Endotoxin, Extracellular polysaccharides, β(1,3)-D-glucan, Pets, Dampness, Indoor, Children, Cohort study

## Abstract

**Background:**

Inflammation is a key factor in the pathogenesis of respiratory diseases. Early life exposure to microbial agents may have an effect on the development of the immune system and on respiratory health later in life.

In the present work we aimed to evaluate the associations between early life microbial exposures, and fractional exhaled nitric oxide (FeNO) at school age.

**Methods:**

Endotoxin, extracellular polysaccharides (EPS) and β(1,3)-D-glucan were measured in living room dust collected at 2–3 months of age in homes of participants of three prospective European birth cohorts (LISA, n = 182; PIAMA, n = 244; and INMA, n = 355). Home dampness and pet ownership were periodically reported by the parents through questionnaires. FeNO was measured at age 8 for PIAMA and at age 10/11 for LISA and INMA. Cohort-specific associations between the indoor microbial exposures and FeNO were evaluated using multivariable regression analyses. Estimates were combined using random-effects meta-analyses.

**Results:**

FeNO at school age was lower in children exposed to endotoxin at age 2–3 months (β -0.05, 95% confidence interval (CI) -0.10;-0.01) and in children with reported dog ownership during the first two years of life (GM ratio 0.82, CI 0.70-0.96). FeNO was not significantly associated with early life exposure to EPS, β(1,3)-D-glucan, indoor dampness and cat ownership.

**Conclusion:**

Early life exposure to bacterial endotoxin and early life dog ownership are associated with lower FeNO at school age. Further studies are needed to confirm our results and to unravel the underlying mechanisms and possible clinical relevance of this finding.

## Background

The first years of life may be a crucial period for the development of the immune system and the onset of allergic and respiratory disorders [1,2]. In the last decade, numerous studies assessed associations between measured microbial agents and related indoor factors and reported respiratory and allergic outcomes in children [3-13]. Only few studies measured early-life microbial exposures and investigated health effects prospectively. They found that exposure to high levels of endotoxin in the first months of life may reduce the risk of eczema in infancy [7]. However, findings regarding wheezing and asthma are inconsistent [5,6,14]. Regarding indoor factors associated with higher indoor microbial agents levels, previous studies observed that early life exposure to indoor mould and dampness increases the risk of asthma [15-17]. However, the direction of the associations between early life exposure to pet ownership and atopy, wheezing and asthma is ambiguous. Some studies found that the presence of a dog in the home during early life attenuated TNF-α production [18] and had a protective effect on atopy and wheezing [19,20]. Other studies observed positive or non-significant associations between home exposure to cats or dogs and atopy, respiratory symptoms and asthma [21-24]. In addition, a systematic review suggested no association with dog ownership with large heterogeneity across studies [25].

Inflammation is a key factor in the pathogenesis of respiratory diseases and FeNO is considered to be a non-invasive biomarker that is associated with eosinophilic airway inflammation [26-28]. High levels of NO in the airways are associated with asthma and other respiratory disorders [29] and with respiratory symptoms and atopy in children [30-33]. In epidemiological studies, using FeNO has the advantage that it is a continuous and easily measureable variable, as well as an objective variable, in contrast to self-reported categorical variables. Several epidemiologic studies showed associations between environmental exposures such as air pollution [34,35], polycyclic aromatic hydrocarbons [36], indoor allergens [37] and other indoor factors [38] and FeNO. However, the effects of early life exposure to microbial agents and indoor factors on FeNO have not been explored. In our study, we assessed the associations of microbial agents (endotoxin, EPS and β(1,3)-D-glucans) in house dust, reported dampness and pet ownership early in life with FeNO in school-aged children in three European birth cohorts participating in the European HITEA project (Health Effects of Indoor Pollutants: Integrating microbial, toxicological and epidemiological approaches).

## Methods

### Study population and design: description of the three birth cohorts

As part of the European HITEA project, the present study includes information from three ongoing European birth cohorts that started between 1996 and 1999: LISA (influence of life-style factors on the development of the immune system and allergies in East and West Germany) in Germany [39], PIAMA (Prevention and Incidence of Asthma and Mite Allergy) in the Netherlands [40]. and INMA (INfancia y Medio Ambiente [Environment and Childhood]) in Spain [41]. Written informed consent was obtained from all parents and the studies were approved by the local ethics committees in each cohort region: the Ethik-Kommission der Bayerischen Landesärztekammer for LISA, the Centrale Commissie Mensgebonden Onderzoek (CCMO), the Medisch Ethische Commissies of the University Medical Center Utrecht, the Erasmus Medical Center Rotterdam, and the Academisch Ziekenhuis Groningen for PIAMA and the Ethics Committee of the Institut Municipal d’Investigació Mèdica – Parc de Salut Mar for INMA. A description of the participating birth cohorts is given in the Additional file 1 and in the paper describing the exposures under study [42]. The present study includes children with measured endotoxin, EPS and/or β(1,3)-D-glucans in living room dust collected during early life and FeNO measurements at school age (182 from LISA, 244 from PIAMA, and 355 from INMA).

### Fractional exhaled Nitric Oxide (FeNO)

FeNO was measured in LISA at 10 years of age, in PIAMA at 8 years of age [43] and in INMA at 10 to 13 years of age, according to the American Thoracic Society guidelines [44] using the NIOX MINO^®^ in LISA and INMA and the NIOX analyser (Aerocrine, Solna, Sweden; http://www.aerocrine.com) in PIAMA. In LISA and INMA children were refrained from eating or drinking one hour before the measurement. They were asked to inhale to near-total lung capacity through the NIOX MINO^®^, and to exhale immediately at a constant flow rate of 50 mL/sec until a NO plateau of at least 3 seconds could be identified during an exhalation of at least 6 seconds. Measurements in both cohorts were performed until a correct FENO measurement was displayed. If necessary, the FENO test was repeated to obtain one acceptable measurement.

In PIAMA, children were instructed to inhale to near-total lung capacity through a NO scrubber (Dräger combination filter, Dräger, Lübeck, Germany), integrated in the NIOX analyzer, and to exhale immediately at a constant flow rate of 50 mL/second, until a NO plateau of at least 2 seconds could be identified during an exhalation of at least 4 seconds. A maximum of 6 attempts were performed to obtain three acceptable FENO measurements. FENO in PIAMA is expressed as the average of the 3 measurements.

In the LISA cohort children who had taken any anti-asthmatic or anti-inflamatory medication did not perform the test. In PIAMA and INMA the use of anti-asthmatic medication (in the past 24 hours in INMA, in the past 48 hours in PIAMA) or anti-inflammatory medication (in the past 24 hours for INMA and PIAMA) was recorded. Four children in PIAMA and 49 in INMA had taken anti-asthmatic or anti-inflammatory medication. In the three birth cohorts, all values were above the LOD. Children did not have any active upper or lower respiratory tract viral infection on the day of the measurement.

### Dust collection, extraction and analyses

Living room dust samples were collected at the child’s age of 2–3 months in the homes of the participants, using vacuum cleaners equipped with ALK filterholders containing a paper filter and the date of sampling was recorded. Samples were collected on living room floors in LISA and PIAMA, and on living room sofa in INMA. Collected samples were stored at -20°C, and analyzed for microbial agents at in the Institute for Risk Assessment Sciences (IRAS, Utrecht, NL). Endotoxin has been determined with the Limulus Amebocyte Lysate test and glucan and EPS with specific enzyme immunoassays as in previous house dust analyses [45]. Levels were expressed in Endotoxin Units (EU), EPS Units (EPSU), and μg of β(1,3)-D-glucan per mL, with as lower limit of detection (LOD) 10 EU/mL (2 EU/mL in INMA), 180 EPSU/mL, and 2 μg/mL, respectively, and converted into concentrations in the original dust samples (in EU/mg, EPSU/mg and μg/mg). Samples with non-detectable amounts of endotoxin, glucans and EPS were assigned a value of 2/3 of the LOD. Detailed information on the dust collection, extraction and analyses and a description of the values below the LOD is shown elsewhere [42].

### Reported indoor factors: dampness at home and cat and dog ownership

Questions about housing characteristics and potential exposures in the home environment were taken from questionnaires administered to the parents from birth to the child’s age of 8 (PIAMA) and 10 years (LISA and INMA). Dampness or visible mould at home was reported by the parents at the child’s ages of 3 months, 1 and 2 years in the three cohorts. We combined these data into a single binary exposure variable on ever reported dampness or visible mould during the first 2 years of life. Cat and dog ownership was reported at birth and at the child’s ages of 1, 2, 4, 6 and 10 (8 in PIAMA) years in the three cohorts. For the statistical analyses, we computed separate 3-category variables for cat and for dog, describing the timing of the first cat/dog ownership. These variables indicated “Never” (no report of ownership), “Ever during the first 2 years of life” (at least one report of ownership in the first 2 years of life), and “Ever after the first 2 years of life, but not during the first 2 years of life” (at least one report of ownership after the first 2 years of life, but no report during the first 2 years) exposure.

### Potential confounders and effect modifiers

The questionnaires administered during pregnancy or at birth and at the child’s ages of 1, 2, 4, 6 and 10 (8 in PIAMA) years included socio-demographic, health and environmental data such as: parental education (low, medium or high); medical data of the parents; parental smoking; housing characteristics including the location of the home (area population density) and moving to another home. Information about the child’s allergic and respiratory health was obtained from parental reports of hay fever, rhinitis, eczema, wheezing, asthma medication and doctor diagnosed asthma. Two new variables, one for asthma and another including reported allergies, were computed and considered as potential effect modifiers. Asthma cases were children with a positive answer to two out of three of the following questions: doctor diagnosed asthma; wheezing; asthma medication, in any year between 4 and10 (8 in PIAMA) years of age. Allergy cases were defined as children with at least one positive answer to questions on hay fever, rhinitis and eczema in any year between 4 and 10 (8 in PIAMA) years of age. Atopy was assessed in 74% of the study population. Specific immunoglobulin E (sIgE) was measured in blood in the three cohorts at different time points: 10 years of age in LISA, 8 years of age in PIAMA and 4 years of age in INMA. In LISA, atopy was defined as sIgE of at least 0.35 kU/l for 'sx1 inhalant mixture’ (timothy, rye, mugwort, house dust mite (*Dermatophagoides pteronyssinus,* Der p), cat, dog and mould mixture). In PIAMA, atopy was defined as sIgE of at least 0.35 kU/l for house dust mite (Der p), cat, dog, birch, *Alternaria alternata* and *Dactylis glomerata*. Finally, atopy in INMA was defined as sIgE ≥0.35U/mL of IgE for Der p, cat or grass/pollen. A list of all the potential confounders evaluated in this study is provided in the Additional file 1.

### Statistical analyses

The distribution of the FeNO, endotoxin, EPS and glucan measurements was right-skewed and therefore their levels were natural log-transformed. Generalized additive models (GAM) [46] were used to assess the functional relationship between the concentrations of microbial agents and FeNO stratified per cohort and in cohort-adjusted pooled analyses. With descriptive purposes, we computed quartiles of endotoxin concentrations and tertiles of EPS and glucan concentrations and added a < LOD category that included samples with microbial agents measurements below the limit of detection. These variables were calculated to show the distribution of FeNO according to the levels of microbial agents. A description of the quantiles is shown in Table E1 of the Additional file 1. Nevertheless, the main analyses are conducted using the microbial agents concentrations as continuous variables.

To assess the adjusted associations between FeNO and the exposure variables by cohort, we performed multivariable linear regression models. Potential confounders were a-priori identified from the literature and selected based on their relationship with FeNO and the exposure variables in the present study (listed in the Additional file 1). The concentration of each microbial agent (endotoxin, EPS and glucans) and target indoor factor (dampness and cat and dog ownership) was included separately in the models. In order to facilitate interpretation, the resulting regression coefficients for categorical variables were back-transformed to their exponential (exp(β)), yielding the ratio of the GM of FeNO of each exposure category vs the reference category. For continuous natural log-transformed variables, we present the coefficients (β) in the tables. To facilitate the interpretation of these coefficients in the text, we transformed the coefficients to (2^β^-1)*100. This transformation allows interpreting the coefficient as the percentage of increase in FeNO when doubling the dose of each microbial agent. In order to assess the possibility of effect modification of studied associations with the report of allergy and atopy, we included interaction terms between the report of allergy and atopy and the exposure variables and we performed stratified analyses. For additional sensitivity analyses, we excluded individuals that had taken anti-asthmatic or anti-inflammatory medication during the 48 or 24 hours prior to the FeNO measurement.

Finally, we performed random-effects meta-analyses including the three cohort-specific estimates. We obtained combined GM ratios and their CI for each microbial agent and indoor factor. Potential heterogeneity between cohorts was examined using the Cochran Q statistics (p-value < 0.05). Data analysis was conducted with STATA SE 10.0 statistical software (Stata Corporation, College Station, TX, USA).

## Results

A description of the socio-demographic, respiratory health and indoor exposure characteristics of the HITEA participants included and not included in this study is shown in Table 1. Children included were not different from those not included regarding most characteristics. Across the participating cohorts, we observed statistically significant differences for all variables except sex and doctor diagnosed asthma. Higher parental education was observed in the LISA cohort, higher asthma prevalence in PIAMA and higher smoking report in INMA compared to the other cohorts. The percentage of children with allergy report was higher in the PIAMA cohort whereas the age at FeNO measurement and FeNO was lower, the percentage of children with atopy was higher in LISA, where children were older at the moment of sIgE measurements (10 years old). Regarding the exposure variables, in INMA, endotoxin concentrations were lower and the prevalence of dampness and dog ownership in the first two years of life was higher. Cat ownership in the first two years of life was higher in PIAMA.

**Table 1:** Description of the HITEA project participants, not included and included in the study

			**LISA**		**PIAMA**		**INMA**	
			**Not included**	**Included**	**Not included**	**Included**	**Not included**	**Included**
			**n = 213**	**n = 182**	**n = 309**	**n = 244**	**n = 126**	**n = 355**
**Population characteristics**								
	**Sex (female): n(%)**		104 (48.8)	76 (41.8)	139 (45)	127 (52.1)	57 (45.2)	177 (49.9)
	**Age at FeNO measurement (years): mean (sd)**		-	10.2 (0.2)	-	8.0 (0.3)	-	11.5 (0.7)
	**FeNO (ppb): GM (Gsd)**		-	14.7 (2.0)	-	10.2 (2.0)	-	12.4 (2.3)
	Asthma (4 to 10 years old*): n (%)		9 (8.9)	10 (6.1)	20 (14.5)	19 (10.6)	33 (10.3)	41 (10.8)
	**Atopy (sIgE ≥0.35U/mL)**^**a**^**: n (%)**		8 (53.3)	80 (47.6)	45 (43.3)	71 (36.8)	7 (14.3)	36 (16.1)
	Reported allergies (4 to 10 years old*): n (%)^**bd**^		50 (39.4)	46 (27.1)	64 (41.6)	86 (39.8)	41 (51.3)	117 (33.9)
	**Parental education: n (%)**^**d**^							
		**Secondary school**	38 (18.4)	20 (11.4)	98 (37.3)	80 (33.3)	38 (31.7)	130 (38.4)
		**Higher than secondary school**	159 (76.8)	148 (84.6)	131 (49.8)	140 (58.3)	12 (10.0)	67 (19.8)
	**Parental asthma: n (%)**		25 (12.3)	21 (11.9)	93 (30.8)	84 (35.0)	17 (14.1)	34 (9.6)
	Parental smoking at the age of 10 years*: n (%)		9 (8.7)	11 (6.4)	9 (6.1)	10 (5.0)	28 (45.2)	115 (35.8)
**Indoor exposures**								
	**Endotoxin concentrations (EU/mg): GM (Gsd)**		22.6 (2.5)	23.5 (2.4)	23.2 (3.7)	20.6 (3.2)	4.0 (6.3)	3.1 (6.4)
	**EPS concentrations (U/mg): GM (Gsd)**		46.6 (2.5)	48.4 (2.1)	29.1 (2.8)	32.9 (3.5)	119.9 (2.7)	116.4 (2.5)
	**Glucan concentrations (ug/mg): GM (Gsd)**		1.9 (1.7)	2.0 (1.7)	1.6 (2.1)	1.8 (2.4)	-	-
	**Dampness at home (first 2 years of life)**^**c**^		74 (35.1)	62 (34.3)	159 (64.9)	121 (54.8)	72 (57.6)	221 (63.3)
	**Cat ownership: n (%)**^**c**^							
		**Ever during the first 2 years of life**	25 (20.2)	23 (13.8)	79 (42)	59 (27.2)	15 (20.3)	68 (19.3)
		**Ever after the first 2 years of life**	17 (13.7)	26 (15.6)	12 (6.4)	19 (8.8)	10 (13.5)	30 (8.5)
	**Dog ownership: n (%)**^**bcd**^							
		**Ever during the first 2 years of life**	16 (13.5)	8 (5.0)	51 (28.5)	31 (14.5)	45 (47.9)	143 (40.9)
		**Ever after the first 2 years of life**	16 (13.5)	16 (10.1)	32 (17.9)	16 (7.5)	19 (20.2)	45 (12.9)

sd: standard deviation; GM: geometric mean; Gsd: geometric standard deviation; FeNO: fractional exhaled nitric oxide; Reported allergies: eczema, rhinitis or hay fever.

*8 years in PIAMA; ^a^LISA n not included = 15 and n included = 168, PIAMA n not included = 104 and n included = 193, INMA n not included = 49 and n included = 224; ^b^p-value < 0.05 of the difference between included and not included in LISA; ^c^p-value < 0.05 of the difference between included and not included in PIAMA; ^d^p-value < 0.05 of the difference between included and not included in INMA.

Geometric means of FeNO were lower in individuals classified in the highest endotoxin quartile in INMA, FeNO was lower in the mid tertile of EPS in LISA and INMA and similar trends were observed for glucans in LISA. Children exposed to cats and to dogs in the first two years had lower FeNO, except for cats exposure in LISA. However the differences were not statistically significant (p-values ≥ 0.05) (Table 2).

**Table 2:** Description of the FeNO (ppb) according to the asthmatic status and exposure per cohort

	**LISA**	**PIAMA**	**INMA**
	**n = 182**	**n = 244**	**n = 355**
	**GM (Gsd)**	**GM (Gsd)**	**GM (Gsd)**
**Asthma**			
**No**	13.7 (1.9)	10.4 (1.9)	11.3 (2.1)
**Yes**	30.3 (2.3)	13.4 (2.0)	27.3 (3.0)
**Endotoxin concentrations (EU/mg)**			
**Q1 (<3.54 EU/mg)**	12.6 (1.4)	7.8 (1.5)	12.3 (2.4)
**Q2 (>3.54-12.4 EU/mg)**	17.2 (1.9)	11.5 (2.2)	13.0 (2.2)
**Q3 (>12.4-30.6 EU/mg)**	14.3 (2.0)	10.1 (1.9)	11.8 (2.1)
**Q4 (>30.6 EU/mg)**	14.4 (2.0)	9.7 (1.8)	11.5 (2.2)
**EPS concentrations (U/mg)**			
**<LOD**	14.2 (2.3)	9.5 (2.0)	10.4 (1.8)
**T1 (<42 U/mg)**	15.2 (2.0)	10.6 (2.0)	11.8 (2.3)
**T2 (>42-101 U/mg)**	13.8 (1.9)	9.9 (1.9)	12.1 (2.1)
**T3 (>101 U/mg)**	16.2 (2.2)	12.5 (1.8)	13.2 (2.4)
**Glucan concentrations (ug/mg)**			
**<LOD**	-	9.4 (2.1)	-
**T1 (<1.55 μg/mg)**	15.8 (2.0)	10.9 (1.9)	-
**T2 (>1.55-2.35 μg/mg)**	13.6 (1.9)	10.3 (1.9)	-
**T3 (>2.35 μg/mg)**	15.4 (2.1)	10.7 (1.8)	-
**Dampness at home**			
**Never**	14.9 (2.0)	10.7 (1.9)	12.9 (2.3)
**Ever during the first 2 years of life**	14.3 (2.1)	10.0 (2.0)	12.1 (2.3)
**Cat ownership**			
**Never**	15.5 (2.0)	10.6 (1.9)	12.7 (2.3)
**Ever during the first 2 years of life**	16.1 (2.1)	9.7 (1.8)	10.8 (2.2)
**Ever after the first 2 years of life**	11.4 (1.7)	9.9 (2.4)	15.1 (2.5)
**Dog ownership**			
**Never**	15.1 (2.0)	10.5 (2.0)	13.3 (2.4)
**Ever during the first 2 years of life**	13.4 (1.6)	9.0 (1.8)	11.6 (2.3)
**Ever after the first 2 years of life**	15.5 (1.6)	10.9 (2.1)	12.3 (2.1)

GM: geometric mean; Gsd: geometric standard deviation; Q: quartile; T: tertile; EPS: extracellular polysaccharides; <LOD: below the limit of detection.

Sex, age at FeNO measurement, asthma, reported allergies, indoor smoking and parental education were associated with FeNO after mutual adjustment (p-values < 0.05) and thus included in the multivariate models. Parental education was also associated with the levels of indoor microbial agents, asthma and indoor smoking with pet ownership, and indoor smoking was additionally associated with reported dampness.

**Figure 1:**
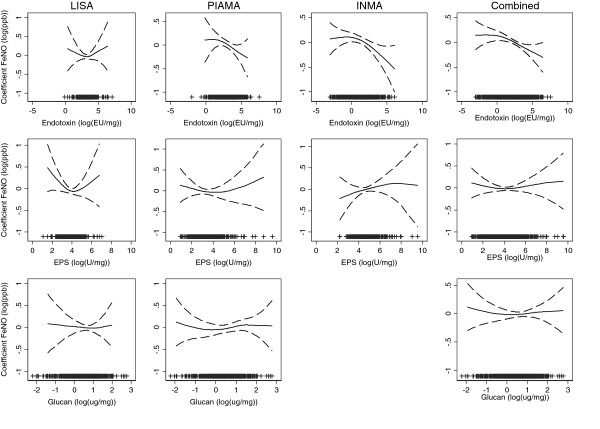
**Smoothed association between natural-log-transformed FeNO coefficients and natural-log-transformed microbial agents concentrations by cohort and pooled.** Adjusted for sex, age of FeNO measurement, asthma, reported allergies (hay fever, rhinitis or eczema), parental smoking at the moment of the FeNO measurement, parental education and season of dust sampling. The Combined graphs were additionally adjusted for cohort.

The smoothed association between FeNO and the microbial agents concentrations, per cohort and combined, showed that the associations were linear (p-values > 0.05 in all cases, Figure 1). The combined random-effects adjusted coefficients for microbial agents concentrations were statistically significant for endotoxin, showing a 3.7% decrease in FeNO when doubling the dose of endotoxin (β = -0.05; 95% CI:-0.10; -0.01). In addition, combined random-effects adjusted GM ratios of FeNO were lower for individuals with reported dog ownership during the first 2 years of life. Association estimates for EPS, glucan, dampness and cat ownership were not statistically significant (p-values ≥ 0.05). P-values for heterogeneity were above 0.1 in all cases (Table 3). These associations were adjusted for sex, age at FeNO measurement, asthma, parental indoor smoking at the time of the FeNO measurement, and parental education. Models including microbial agents measurements were additionally adjusted for season of dust sampling. Additional adjustment for other potential confounders such as season of FeNO measurement, asthma or anti-inflammatory medication prior to the FeNO measurement, outdoor NO_2_ at school age or ever moving to another home did not modify the effect estimates. Additional adjustment for atopy resulted in an attenuation of the effects. Also, the effect estimates for endotoxin and early life pet ownership became statistically non-significant. The effect estimates for endotoxin and early life dog ownership after these additional adjustments are shown in Table E2 in the Additional file 1.

**Table 3:** FeNO adjusted associations with natural-log-transformed microbial agent concentrations, reported home dampness and pet ownership

				**Combined (random effects)**		
	**LISA**	**PIAMA**	**INMA**	**Total**	**Not reported allergies**	**Reported allergies**
	**β (95% CI)**	**β (95% CI)**	**β (95% CI)**	**β (95% CI)**	**β (95% CI)**	**β (95% CI)**
**Microbial agents concentrations**						
**Endotoxin (log(EU/mg))**	0.01 (-0.11; 0.13)	-0.08 (-0.17; 0.01)	-0.06 (-0.11; 0.00)	-0.05 (-0.10; -0.01)	-0.06 (-0.11; -0.01)	-0.06 (-0.14; 0.03)
**EPS (log(U/mg))**	-0.03 (-0.16; 0.11)	0.02 (-0.07; 0.11)	0.06 (-0.05; 0.17)	0.02 (-0.04; 0.08)	0.07 (0.00; 0.15)	0.08 (-0.05; 0.20)
**Glucan (log(μg/mg))**	-0.07 (-0.28; 0.15)	0.02 (-0.10; 0.13)	-	0.00 (-0.10; 0.10)	0.00 (-0.21; 0.21)	0.04 (-0.15; 0.24)
	**GM ratio (95%CI)**	**GM ratio (95%CI)**	**GM ratio (95%CI)**	**GM ratio (95%CI)**	**GM ratio (95%CI)**	**GM ratio (95%CI)**
**Dampness at home**						
**Never**	1	1	1	1	1	1
**Ever during the first 2 years of life**	1.01 (0.80-1.26)	0.89 (0.73-1.1)	1.03 (0.85-1.24)	0.97 (0.87-1.10)	0.93 (0.81-1.06)	1.14 (0.8-1.63)
**Cat ownership**						
**Never**	1	1	1	1	1	1
**Ever during the first 2 years of life**	0.97 (0.71-1.33)	0.89 (0.70-1.13)	0.83 (0.65-1.06)	0.89 (0.76-1.03)	0.98 (0.78-1.22)	0.72 (0.51-1.04)
**Ever after the first 2 years of life**	0.87 (0.64-1.19)	0.96 (0.65-1.43)	1.22 (0.88-1.69)	1.01 (0.82-1.24)	1.11 (0.90-1.37)	0.83 (0.52-1.32)
**Dog ownership**						
**Never**	1	1	1	1	1	1
**Ever during the first 2 years of life**	1.04 (0.65-1.68)	0.89 (0.66-1.20)	0.75 (0.62-0.92)	0.82 (0.70-0.96)	0.88 (0.74-1.04)	0.69 (0.51-0.94)
**Ever after the first 2 years of life**	0.83 (0.57-1.21)	1.18 (0.80-1.76)	1.00 (0.75-1.33)	0.99 (0.81-1.21)	1.02 (0.82-1.27)	1.28 (0.5-3.29)

Adjusted for sex, age of FeNO measurement, asthma, reported allergies, parental smoking at the moment of the FeNO measurement, and parental education. Models including endotoxin, EPS and glucan measurements were additionally adjusted for season of dust sampling. Models stratified by reported allergies were not adjusted for reported allergies. All the heterogeneity tests were not significant (p-values ≥ 0.05).

The association estimates obtained for each exposure variable were homogeneous across cohorts (heterogeneity p-values ≥ 0.05). Stratification by parental report of child allergy did not show significant differences (see Table E3 in the Additional file 1). Moreover, adjusted GM ratios for the non-asthmatic population (Table E4 in the Additional file 1) were similar to those presented in Table 3. Finally, the exclusion of individuals who had taken anti-asthmatic or anti-inflammatory medication in the 48 or 24 hours previous to the FeNO measurement did not modify our results.

## Discussion

Higher endotoxin concentrations and presence of dogs in the home during early life were associated with lower FeNO at school age. We investigated the association of FeNO as a potential biomarker of eosinophilic airway inflammation and microbial exposure variables, independently of any association with asthma or atopy. Our results do not suggest association with disease, but pertain to potential long-term effects of early life exposures on the immune system. Experimental animal studies previously showed that endotoxin and allergen exposure during pregnancy may modulate airway inflammation in offspring later in life, resulting in reduced allergen-induced immune and airway responses [47,48].

We observed that endotoxin exposure levels and dog ownership early in life were associated with lower FeNO. No associations were found for EPS and glucan exposure, cat ownership and reported home dampness. This is in line with previous epidemiological studies focusing on the health effects of residential exposure to microbial agents and pets in children, evaluating associations with respiratory symptoms, asthma and atopy. Most previous studies reported that endotoxin may protect against allergen sensitization, atopic eczema, wheezing and asthma later in life [6,7,49,50]. Regarding the exposure to fungal agents such as EPS or glucans, our study did not find statistically significant associations with FeNO. These results are in line with those obtained in previous studies on the association of EPS and glucan levels with respiratory symptoms [6,21]. However, previous studies have reported increased risks of asthma during childhood in association with mould exposure [15-17]. In our study, we did not find an association between early life reports of home dampness and FeNO at school age. The lack of associations in our study could be explained by the fact that our exposure was parental-reported dampness and not objective observation. However, results on previous studies regarding early life exposure to dogs are in line with our findings. Dog ownership was suggested to be inversely associated with allergic sensitization and respiratory symptoms [18,19,50].

Asthma and atopy are known to be associated with high FeNO in children [31,51,52]. Moreover, children with persistent and late onset wheezing phenotypes are more likely to show higher FeNO at school age [30] and have higher risk of atopy and asthma [53,54]. FeNO is a test that may be used to support the diagnosis of asthma, and its role in asthma monitoring remains to be defined [55]. The association between the biochemical events involved in the release of NO in the airways and the inflammatory mechanisms needs to be further studied [56], but, at present, FeNO is, a extensively studied biomarker of airway inflammation and its non-invasive nature makes it suitable for epidemiological studies [27,44,55,57]. In line with the previous studies, FeNO in our study was higher in asthmatic, allergic and atopic children. We did not find differences in the associations between endotoxin and dog exposures and FeNO according to the report of asthma or allergy. However, the additional adjustment for atopy resulted in a decrease in the magnitude of the effect estimate of both exposures on FeNO while the direction of the effect remained the same. In addition, we considered the possibility that the early life exposures evaluated in our study were associated with FeNO through an association with atopy and not through airway inflammation. The statistical analyses with atopy as outcome did not show significant associations between atopy and endotoxin concentrations or early life dog ownership (data not shown).

However, caution is required when interpreting the results including information on atopy. Specific IgE levels in blood were not available in 26% of our study population and they were measured at different time points in each cohort (at the age of the FeNO measurement in LISA and PIAMA and at 4 years of age in INMA).

Our longitudinal study is the first epidemiologic study assessing the long-term effects of early life exposure to indoor microbial agents and related indoor factors on FeNO measured at school age. Our study includes data from subpopulations of three European birth cohorts in three geographically spread locations and benefits from the availability of objective measurements of exposure during early life (2–3 months) and objective measurements of airway inflammation at school age, in addition to reported information periodically collected through questionnaires administered from pregnancy or birth to school age.

A few limitations must be considered when interpreting our results. The study populations involved in the HITEA project were selected differently depending on the cohort. Only subsamples of LISA and PIAMA cohorts were included in the project. Inclusion was based on the availability of dust samples. In LISA, children included in the present study never moved to another home since early life. In PIAMA, the HITEA project includes children from the intervention study (high risk children of allergic mothers). In addition, several differences existed between cohorts in subject characteristics and dust sampling and analyses. In descriptive analyses FeNO values and age were lowest in PIAMA, the cohort with the highest number of allergic children. Results from multivariable regression analyses showed that FeNO, after age- and reported allergy adjustment, were 40% lower in INMA compared to PIAMA (GM ratio = 0.6; 95% CI: 0.4-0.9), and that FeNO in PIAMA children was not different from that in LISA children (GM ratio = 0.9; 95% CI: 0.7-1.2). Regarding dust sampling and analyses, INMA dust samples were taken from sofa instead of floor because of the lack of carpets or rugs in the homes of the Menorca island. Furthermore, INMA samples were extracted in borate buffered saline (BBS) for earlier analysis of house dust mite allergens, which is not the standard extraction fluid used for endotoxin analyses, additionally the lack of heat-extraction impeded glucan quantification from these samples.

Overall, the association estimates differed somewhat by cohort. This might be explained by differences in inclusion criteria, in participants characteristics and in dust sampling and analyses. For these reasons, we explored to what extent our results are driven by only one or two cohorts by performing sensitivity analyses where we excluded one cohort at a time from the meta-analysis. The results from this sensitivity analyses showed that the combined coefficients of endotoxin exposure and for early life dog ownership are statistically significant when excluding LISA from the meta-analyses but not significant when excluding PIAMA or INMA. Nevertheless, the magnitude and direction of the coefficients did not change. The number of individuals included in the LISA cohort is lower than in the other cohorts. Therefore, the lack of statistical significance in the estimates when including LISA and only one of the other participating cohorts may be related to the statistical power and with the narrow range of exposures in LISA. In addition, LISA homes were located in a high density area (Munich city) while in the PIAMA and INMA cohorts the density of the area where homes were located varied. The density of the area where participants live may influence their behaviour regarding pet ownership and the levels of indoor microbial agents. This may also explain the differences observed in the coefficients for endotoxin exposure and dog ownership.

Apart from the differences across cohorts, other limitations related with the associations between the exposures, the outcome and the respiratory symptoms and allergy should be considered. FeNO is associated with atopy and asthma [26,27,31,51,52]. On the other hand, asthma and atopy may be associated with early life exposure to microbial agents and related indoor factors [5,6,15]. In order to assess the possibility of effect modification by reported allergy or atopy, we included interaction terms between the exposure variables in the models and reported allergy and atopy. Associations for the interaction terms between reported allergy or atopy and endotoxin and dog ownership were not statistically significant (p-values > 0.1). Moreover, stratified analyses did not show differences in the effect estimates according to the disease group. However, because of the limited power after stratification, these results must be cautiously interpreted.

Furthermore, dog ownership is associated with higher levels of indoor endotoxins. In order to evaluate the independent effects of these two indoor factors on FeNO, we calculated the mutually adjusted coefficients for dog ownership and endotoxin concentrations. These coefficients were not different from those shown in Table 3. Therefore, we may assume that the effects observed for the exposures to endotoxin and dog ownership on FeNO are independent.

Finally, because the differences in FeNO across the different levels of exposure were small we must consider the possibility that our findings are due to a statistical association without a biological basis. Nevertheless, the consistency between our findings with dog ownership only in the first two years of life and endotoxin exposure supports the biological basis of our findings.

## Conclusions

Exposure to high concentrations of bacterial endotoxin in the first months of life and dog ownership during the first two years of life, but not exposure to fungal microbial agents or home dampness, was associated with lower FeNO at school age. Further studies are needed to assess how this association is biologically explained, and what the clinical relevance could be.

## Abbreviations

EPS: Extracellular polysaccharides; FeNO: Fractional exhaled nitric oxide; NO: Nitric oxide; GM: Geometric mean; Gsd: Geometric standard deviation; sd: standard deviation; CI: 95% confidence interval; EU: Endotoxin units; EPSU: Extracellular polysaccharides units; LOD: Limit of detection; BBS: Borate buffered saline.

## Competing interests

The authors declare that they have no competing interests.

## Authors’ contributions

LC performed the statistical analyses and drafted the manuscript. LC, CT and IMW were responsible for the study design. RGE was the data manager. AH was the Principal Investigator of the HITEA project and JS was the Principal Investigator of this study within the HITEA project. All other authors critically reviewed the manuscript and approved the final version of the manuscript for submission. All authors read and approved the final manuscript.

## Supplementary Material

Additional file 1 Additional description of the study population, methods and results. Click here for file
