# Supplementary material for: Early life microbial exposure and fractional exhaled nitric oxide in school-age children: a prospective birth cohort study
Source: Environ Health. 2013 Dec 2;12:103. doi: 10.1186/1476-069X-12-103 (PMC3883521; doi:10.1186/1476-069X-12-103)
Supplement: Additional file 1 — Additional description of the study population, methods and results. [file 1476-069X-12-103-S1.doc]

**Description of the study population**

**LISA**

In the city of Munich (Germany), a total number of 1467 neonates were recruited between December 1997 and January 1999. Living room floor dust samples were collected at the child’s age of 3 months and information regarding child’s health was obtained through yearly questionnaires and health exams from birth to the age of 4 and at the child’s ages of 6 and 10 years. The HITEA project included a randomly selected sample of those LISA participants with a high response rate from birth to the age of 10 years and with living room dust samples available (n=448) and microbial agents concentrations could be measured in 395 samples. From the selected children, 182 had FeNO measurements at 10 years of age.

**PIAMA**

The PIAMA study is conducted in the Netherlands and consists of a natural history cohort and an intervention study. Early life microbial exposure was only assessed in the intervention study, as described previously.[1] The HITEA study thus included children from the intervention study only. In this part of the study, mothers with self-reported allergies, asthma or both were invited to participate. Of the 810 participants: one group (intervention group) was supplied with mite-impermeable mattress and pillow covers and the other (placebo group) was supplied with placebo cotton covers for use both on the infants’ and their parents’ beds. Samples for microbial analysis were obtained from 696 participants, nicely divided over the intervention (51%) and control (49%) group. Living room floor dust samples were taken at the age of 2-3 months and microbial agents concentrations could be measured in 553 samples. Information regarding child’s health was obtained through yearly questionnaires and health exams from birth to the age of 4 and at the child’s ages of 6, and 8 years. FeNO was available for 244 children aged approximately 8 years and with early life dust samples.

**INMA**

In Menorca island (Spain), all pregnancies from the general population were selected at the third trimester between September 1997 and January1999 (n=486). Living room sofa dust samples were taken at the age of 2-3 months. Information regarding child’s health was obtained through yearly questionnaires and health exams from birth to the age of 4 and at the child’s ages of 6 and 10 years. All participants in the INMA-Menorca cohort with living room dust samples were included in the HITEA project (n=481). FeNO was measured in 355 participants aged 10 to 13 years.

**Potential confounders evaluated in our study:**

- Sex.
- Age at FeNO measurement.
- Reported allergies (hay fever, rhinitis and eczema).
- Parental smoking at the time of the FeNO measurement.
- Parental education.
- Season of dust collection (winter, spring, summer or fall).
- Ever moving to another home between the dust sampling (early life) and the FeNO measurement.
- Atopy: specific IgE (see methods section).
- Having cat(s) or dog(s) in the home at the age when measurement of FeNO took place
- Modelled annual average levels of NO_2_ at the home address at the age when FeNO measurement took place. These levels were obtained from land-use regression models described in detail elsewhere [2].
- Season at the time of the FeNO measurement (winter, spring, summer or fall).
- Use of asthma or anti-inflammatory medication in the 24hr or 48hr prior to the FeNO measurement.

**References**

1. Douwes J, van Strien R, Doekes G, Smit J, Kerkhof M, Gerritsen J, Postma D, de Jongste J, Travier N, Brunekreef B. Does early indoor microbial exposure reduce the risk of asthma? The Prevention and Incidence of Asthma and Mite Allergy birth cohort study. *J. Allergy Clin. Immunol.* 2006; 117: 1067–1073.

2. Beelen R, Hoek G, Vienneau D, Eeftens M, Dimakopoulou K, Pedeli X, Tsai M-Y, Künzli N, Schikowski T, Marcon A, Eriksen KT, Raaschou-Nielsen O, Stephanou E, Patelarou E, Lanki T, Yli-Tuomi T, Declercq C, Falq G, Stempfelet M, Birk M, Cyrys J, von Klot S, Nádor G, Varró MJ, Dėdelė A, Gražulevičienė R, Mölter A, Lindley S, Madsen C, Cesaroni G, et al. Development of NO2 and NOx land use regression models for estimating air pollution exposure in 36 study areas in Europe – The ESCAPE project. *Atmos. Environ.* 2013; 72: 10–23.

Table E1. Description (number and percentage) of the quantiles of microbial agents concentrations in the study population. (Quantiles are based on the exposure distribution of the total population with dust samples)

|  |  | **LISA** | **PIAMA** | **INMA** | **Total** |
| --- | --- | --- | --- | --- | --- |
|  |  | **n=182** | **n=244** | **n=355** | **n=781** |
|  |  | **n(%)** | **n(%)** | **n(%)** | **n(%)** |
| **Endotoxin concentrations (EU/mg)** | |  |  |  |  |
|  | **Q1 (<3.54 EU/mg)** | 4 (2.2) | 12 (5.2) | 174 (50.7) | 190 (25.1) |
|  | **Q2 (>3.54-12.4 EU/mg)** | 30 (16.5) | 66 (28.2) | 93 (27.1) | 189 (24.9) |
|  | **Q3 (>12.4-30.6 EU/mg)** | 82 (45.0) | 67 (28.8) | 41 (12.0) | 190 (25.1) |
|  | **Q4 (>30.6 EU/mg)** | 66 (36.3) | 88 (37.8) | 35 (10.2) | 189 (24.9) |
| **EPS concentrations (U/mg)** | |  |  |  |  |
|  | **<LOD** | 7 (3.9) | 69 (30.1) | 10 (3.0) | 86 (11.5) |
|  | **T1 (<42 U/mg)** | 70 (38.5) | 69 (30.1) | 31 (9.1) | 170 (22.7) |
|  | **T2 (>42-101 U/mg)** | 77 (42.2) | 60 (26.2) | 112 (33.0) | 249 (33.1) |
|  | **T3 (>101 U/mg)** | 28 (15.4) | 31 (13.6) | 186 (54.9) | 245 (32.7) |
| **Glucan concentrations (ug/mg)** | |  |  |  |  |
|  | **<LOD** | 0 (0) | 92 (40.4) | - | 92 (22.3) |
|  | **T1 (<1.55 μg/mg)** | 47 (25.8) | 59 (25.9) | - | 106 (25.9) |
|  | **T2 (>1.55-2.35 μg/mg)** | 75 (41.2) | 31 (13.6) | - | 106 (25.9) |
|  | **T3 (>2.35 μg/mg)** | 60 (33.0) | 46 (20.1) | - | 106 (25.9) |

Q: quartile; T: tertile, EPS: extracellular polysaccharides, <LOD: below the limit of detection

Table E2. Fractional exhaled nitric oxide adjusted combined random effects associations with natural log transformed endotoxin concentrations measured during early life and with dog ownership in the first 2 years of life after additional adjustment for other potential confounders (season of FeNO measurement, asthma medication, outdoor NO^2^, specific IgE and ever moving to another home since birth).

|  | **Endotoxin (log-EU/mg)** | **Dog ownership during the first 2 years of life** |
| --- | --- | --- |
|  | **coef (95%CI)** | **GM (95%CI)** |
| **Season of FeNO measurement** | -0.04 (-0.09; 0.00) | 0.83 (0.71; 0.97) |
| **Asthma medication at FeNO measurement** | -0.05 (-0.09; -0.01) | 0.83 (0.70; 0.98) |
| **Outdoor NO^2^ at school age** | -0.04 (-0.09; 0.00) | 0.83 (0.71; 0.97) |
| **Specific IgE ≥0.35U/mL (Der p, cat or grass/pollen)** | -0.02 (-0.06; 0.03) | 0.86 (0.73; 1.01) |
| **Ever moved to another home** | -0.05 (-0.09; 0.00) | 0.78 (0.66; 0.93) |

Adjusted for sex, age of FeNO measurement, reported allergies, parental smoking at the moment of the FeNO measurement, and parental education. Models including endotoxin measurements were additionally adjusted for season of dust sampling.

Table E3. Fractional exhaled nitric oxide adjusted associations with natural log transformed microbial agent concentrations, with reported home dampness and with pet ownership per cohort stratified by reported allergy (ever rhinitis, hay fever, or eczema).

|  |  | **LISA** | | **PIAMA** | | **INMA** | |
| --- | --- | --- | --- | --- | --- | --- | --- |
|  |  | **Not reported allergies** | **Reported allergies** | **Not reported allergies** | **Reported allergies** | **Not reported allergies** | **Reported allergies** |
|  |  | **n=124** | **n=46** | **n=130** | **n=86** | **n=228** | **n=117** |
|  | | **β (95%CI)** | **β (95%CI)** | **β (95%CI)** | **β (95%CI)** | **β (95%CI)** | **β (95%CI)** |
| **Microbial agents concentrationsa** | |  |  |  |  |  |  |
|  | **Endotoxin (log(EU/mg))** | -0.06 (-0.20; 0.08) | 0.23 (-0.06; 0.51) | -0.03 (-0.13; 0.07) | -0.16 (-0.32; 0.00) | -0.07 (-0.13; 0.00) | -0.03 (-0.13; 0.08) |
|  | **EPS (log(U/mg))** | 0.03 (-0.12; 0.19) | -0.11 (-0.45; 0.23) | 0.04 (-0.07; 0.17) | 0.04 (-0.15; 0.23) | 0.13 (0.01; 0.27) | -0.07 (-0.27; 0.13) |
|  | **Glucan (log(μg/mg))** | -0.13 (-0.39; 0.12) | -0.09 (-0.55; 0.36) | 0.08 (-0.05; 0.22) | -0.08 (-0.30; 0.14) | - | - |
|  | | **GM ratio (95%CI)** | **GM ratio (95%CI)** | **GM ratio (95%CI)** | **GM ratio (95%CI)** | **GM ratio (95%CI)** | **GM ratio (95%CI)** |
| **Dampness at home** | |  |  |  |  |  |  |
|  | **Never** | 1 | 1 | 1 | 1 | 1 | 1 |
|  | **Ever during the first 2 years of life** | 0.97 (0.75-1.25) | 1.17 (0.71-1.93) | 0.95 (0.76-1.19) | 0.83 (0.55-1.26) | 0.85 (0.67-1.09) | 1.49 (1.04-2.13) |
| **Cat ownership** | |  |  |  |  |  |  |
|  | **Never** | 1 | 1 | 1 | 1 | 1 | 1 |
|  | **Ever during the first 2 years of life** | 1.03 (0.74-1.43) | 0.93 (0.31-2.73) | 1.14 (0.88-1.46) | 0.54 (0.32-0.89) | 0.78 (0.58-1.06) | 0.88 (0.56-1.39) |
|  | **Ever after the first 2 years of life** | 0.99 (0.72-1.37) | 0.55 (0.18-1.70) | 1.19 (0.77-1.84) | 0.65 (0.31-1.35) | 1.22 (0.83-1.79) | 1.18 (0.63-2.24) |
| **Dog ownership** | |  |  |  |  |  |  |
|  | **Never** | 1 | 1 | 1 | 1 | 1 | 1 |
|  | **Ever during the first 2 years of life** | 1.05 (0.67-1.65) | - | 0.95 (0.69-1.31) | 0.73 (0.42-1.28) | 0.79 (0.63-1.01) | 0.68 (0.47-0.98) |
|  | **Ever after the first 2 years of life** | 1.03 (0.64-1.64) | 0.67 (0.33-1.37) | 0.99 (0.69-1.42) | 6.69 (1.64-27.28) | 1.05 (0.73-1.49) | 0.90 (0.54-1.49) |

Adjusted for sex, age of FeNO measurement, asthma, parental smoking at the moment of the FeNO measurement, and parental education. Models including microbial agents measurements were additionally adjusted for season of dust sampling.

Table E4. Fractional exhaled nitric oxide adjusted associations with natural log transformed microbial agent concentrations, with reported home dampness and with pet ownership per cohort in children without reported asthma.

|  |  | **LISA** | **PIAMA** | **INMA** | **Combined (random effects)** |
| --- | --- | --- | --- | --- | --- |
|  |  | **β (95%CI)** | **β (95%CI)** | **β (95%CI)** | **β (95%CI)** |
| **Microbial agents concentrationsa** | |  |  |  |  |
|  | **Endotoxin (log(EU/mg))** | -0.02 (-0.14; 0.10) | -0.06 (-0.15; 0.03) | -0.05 (-0.1; 0.01) | -0.05 (-0.09; 0.00) |
|  | **EPS (log(U/mg))** | -0.05 (-0.18; 0.09) | -0.02 (-0.12; 0.08) | 0.08 (-0.03; 0.18) | 0.01 (-0.06; 0.08) |
|  | **Glucan (log(μg/mg))** | -0.09 (-0.30; 0.13) | 0.01 (-0.11; 0.13) | - | -0.02 (-0.12; 0.09) |
|  |  | **GM ratio (95%CI)** | **GM ratio (95%CI)** | **GM ratio (95%CI)** | **GM ratio (95%CI)** |
| **Dampness at home** | |  |  |  |  |
|  | **Never** | 1 | 1 | 1 | 1 |
|  | **Ever during the first 2 years of life** | 1.06 (0.85-1.33) | 0.89 (0.72-1.1) | 0.97 (0.8-1.18) | 0.97 (0.86-1.09) |
| **Cat ownership** | |  |  |  |  |
|  | **Never** | 1 | 1 | 1 | 1 |
|  | **Ever during the first 2 years of life** | 0.99 (0.73-1.34) | 0.92 (0.71-1.19) | 0.9 (0.70-1.17) | 1.00 (0.82-1.21) |
|  | **Ever after the first 2 years of life** | 0.92 (0.68-1.26) | 0.97 (0.65-1.44) | 1.12 (0.8-1.56) | 0.84 (0.72-0.98) |
| **Dog ownership** | |  |  |  |  |
|  | **Never** | 1 | 1 | 1 | 1 |
|  | **Ever during the first 2 years of life** | 1.07 (0.68-1.69) | 0.88 (0.65-1.21) | 0.78 (0.64-0.95) | 0.84 (0.72-0.98) |
|  | **Ever after the first 2 years of life** | 1.00 (0.68-1.48) | 1.17 (0.79-1.74) | 1.08 (0.81-1.44) | 1.08 (0.89-1.32) |

Adjusted for sex, age of FeNO measurement, reported allergies, parental smoking at the moment of the FeNO measurement, and parental education. Models including microbial agents measurements were additionally adjusted for season of dust sampling.
